# Imatinib and Nilotinib Inhibit Hematopoietic Progenitor Cell Growth, but Do Not Prevent Adhesion, Migration and Engraftment of Human Cord Blood CD34^+^ Cells

**DOI:** 10.1371/journal.pone.0052564

**Published:** 2012-12-20

**Authors:** Ludovic Belle, France Bruck, Jacques Foguenne, André Gothot, Yves Beguin, Frédéric Baron, Alexandra Briquet

**Affiliations:** 1 Hematology Research Unit, Giga-I^3^, University of Liège, Liège, Belgium; 2 Department of Laboratory Hematology and Immuno-Hematology, Centre hospitalier universitaire de Liège, Liège, Belgium; 3 Department of Medicine, Division of Hematology, Centre hospitalier universitaire de Liège, Liège, Belgium; New York Medical College, United States of America

## Abstract

**Background:**

The availability of tyrosine kinase inhibitors (TKIs) has considerably changed the management of Philadelphia chromosome positive leukemia. The BCR-ABL inhibitor imatinib is also known to inhibit the tyrosine kinase of the stem cell factor receptor, c-Kit. Nilotinib is 30 times more potent than imatinib towards BCR-ABL *in vitro*. Studies in healthy volunteers and patients with chronic myelogenous leukemia or gastrointestinal stromal tumors have shown that therapeutic doses of nilotinib deliver drug levels similar to those of imatinib. The aim of this study was to compare the inhibitory effects of imatinib and nilotinib on proliferation, differentiation, adhesion, migration and engraftment capacities of human cord blood CD34^+^ cells.

**Design and Methods:**

After a 48-hour cell culture with or without TKIs, CFC, LTC-IC, migration, adhesion and cell cycle analysis were performed. In a second time, the impact of these TKIs on engraftment was assessed in a xenotransplantation model using NOD/SCID/IL-2Rγ (null) mice.

**Results:**

TKIs did not affect LTC-IC frequencies despite *in vitro* inhibition of CFC formation due to inhibition of CD34^+^ cell cycle entry. Adhesion of CD34^+^ cells to retronectin was reduced in the presence of either imatinib or nilotinib but only at high concentrations. Migration through a SDF-1α gradient was not changed by cell culture in the presence of TKIs. Finally, bone marrow cellularity and human chimerism were not affected by daily doses of imatinib and nilotinib in a xenogenic transplantation model. No significant difference was seen between TKIs given the equivalent affinity of imatinib and nilotinib for KIT.

**Conclusions:**

These data suggest that combining non-myeloablative conditioning regimen with TKIs starting the day of the transplantation could be safe.

## Introduction

The Philadelphia (Ph) chromosome results from a reciprocal translocation between chromosomes 9 and 22 resulting in a chimeric *Bcr*-*Abl* gene. The Ph chromosome translocation is present in 95% and 20–30% of patients with chronic myeloid leukemia (CML) and acute lymphoblastic leukemia (ALL), respectively [1]. BCR-ABL proteins possess a constitutive tyrosine kinase activity and play a key role in signaling pathways resulting in the malignant phenotype of hematopoietic stem cells (HSC).

Imatinib (STI571, imatinib, Glivec®; Novartis Pharmaceuticals) is a competitive inhibitor of ATP for binding to BCR-ABL [2] that induces apoptosis in BCR-ABL dependent cells. As a tyrosine kinase inhibitor (TKI), imatinib is not specific towards BCR-ABL, but also inhibits several other kinases including c-Kit, PDGFR, DDR and Abl [3]–[5]. Recently, nilotinib (AMN 107, Tasigna®; Novartis Pharmaceuticals) has been developed with the aim of increasing both potency and selectivity towards BCR-ABL [6]. Nilotinib markedly differs from imatinib in its interactions with the BCR-ABL protein [5], [6] and is 30 times more potent than imatinib against the BCR-ABL *in vitro* activity in several Ph^+^ cell lines [7] and is active against most imatinib-resistant BCR-ABL mutations, but not against the T315I mutant [8], [9].

Allogeneic hematopoietic stem cell transplantation (allo-HSCT) is a potentially curative treatment for patients with Ph^+^ ALL. The majority of older adults with ALL are candidates for a reduced-intensity or a non-myeloablative conditioning regimen [10], [11]. Combining allo-HSCT with TKIs could maximize antileukemic activity against Ph chromosome-positive leukemias [12]. In addition to BCR-ABL, imatinib and nilotinib inhibit c-Kit, the receptor for the stem cell factor (SCF). KIT plays an important role in stem cell biology suppressing apoptosis, inducing cell cycle entry [13], promoting colony growth [14], mediating stem cell self-renewal *in vivo* [15], regulating cell adhesion to fibronectin [16] and mediating chemokinetic and chemotactic signals [17].

However, the impacts of imatinib and nilotinib on HSCs during the post-transplantation period are unknown. Reconstitution of a fully functional hematopoietic system is critical for transplantation outcomes. We have previously shown that imatinib inhibits progenitor cell growth *in vitro,* but does not interfere with engraftment of human hematopoietic stem cells in a xenogenic transplantation model [18]. However, very little information on the toxicity of nilotinib on normal hematopoiesis is available and its effects on HSC engraftment are not known. In this study, we have tested, both *in vitro* and *in vivo*, the inhibitory effects of imatinib and nilotinib on proliferation, differentiation and engraftment capacities of human cord blood CD34^+^ HSCs.

## Materials and Methods

### Isolation of Cord Blood CD34^+^ Cells

After written informed consent of the mother, cord blood was collected according to the standard procedures of the Cord Blood Bank of the University Hospital of Liège. Mononuclear cells were isolated by centrifugation for 40 minutes at room temperature with Ficoll Paque™ plus density gradient (GE Healthcare, Uppsala, Sweden) and washed twice in phosphate-buffered-saline (PBS) (Lonza, Verviers, Belgium) supplemented with 1% Penicillin/Streptomycin (P/S) (Lonza).

CD34^+^ hematopoietic stem cells were isolated by magnetic separation according to the manufacturer’s instructions (Miltenyi Biotech, Gladbach, Germany). First, cells were incubated during 30 minutes at 4°C with a primary anti-CD34 antibody. Cells were washed with PBS+P/S 1% and incubated for 30 minutes at 4°C with a secondary antibody coupled to magnetic beads. Cells were washed in PBS+P/S 1% and passed twice through a MS column (Miltenyi Biotech). CD34^+^ cells were collected after elution of unlabeled cells through the column. Cells were counted with Trypan Blue, washed in PBS and frozen in Fetal Bovine Serum (FBS)+Dimethylsulfoxide (DMSO) 10% (Vel, Leuven, Belgium).

The purity of the CD34^+^ cells was assayed by flow cytometry. A total of 50,000 collected cells were labeled for 30 minutes at 4°C with an allophycoerythrin (APC) conjugated anti-CD34 antibody (BD Biosciences, Erembodegem, Belgium) or with the isotype-matched control (BD Biosciences). Cells were washed twice with PBS+P/S 1% and resuspended in PBS+Formaldehyde 1%. Data acquisition was carried out on a FACSCanto II flow cytometer (BD Biosciences). In all experiments, the percentage of CD34^+^ cells in the starting cell population was higher than 95%.

### Western Blot

Human CD34^+^ cells were thawed in Iscove’s MDM (IMDM) (Lonza) supplemented with bovine serum albumin, insulin, transferrin (BIT) 20% (Stem Cell Technologies, Grenoble, France)+P/S 1% and washed with PBS+P/S 1%. Cells were counted with Trypan Blue and resuspended in IMDM+BIT 20%+P/S 1% at a concentration of 10,000,000 cells/mL. A total of 1,000,000 CD34^+^ cells (100 µL) were seeded in 2.4 mL of IMDM+BIT 20%+P/S 1% supplemented with SCF (100 ng/mL), TPO (50 ng/mL) and FLT-3 (100 ng/mL) (PeproTech, Neuilly-Sur-Seine, France). TKIs were added from a stock solution of 10 mM in DMSO to the medium at a final concentration of 1 or 5 µM. Cells were incubated for 48 hours and then collected and lysed. Total proteins were separated by sodium dodecyl sulfate-polyacrylamide gel electrophoresis. Lysis buffer contained 25 mM Hepes, 150 mM NaCl, 0.5% Triton X-100, 10% glycerol, 1 mM dithiothreitol, phosphatase inhibitors (25 mM β-glycerophosphate, 1 mM Na3VO4, 1 mM NaF) and complete protease inhibitor mixture (Roche Applied Science, Vilvoorde, Belgium). Polyvinylidene fluoride membrane was incubated with 1∶1000 rabbit anti-human phospho-c-Kit (Cell Signaling Technology, Leiden, The Netherlands). The membrane was then incubated with anti-rabbit horseradish peroxidase antibody at 1∶2000 (GE Healthcare, Diegem, Belgium). Goat anti-human actin conjugated to horseradish peroxidase (Santa Cruz Biotechnology, Heidelberg, Germany) was used at 1∶400. Actin signal was used as an internal standard. Finally, the blot was developed using ECL Western Blot detection system (GE Healthcare).

### Colony-forming Cell Assay

CD34^+^ cells were thawed in Iscove’s MDM (IMDM) (Lonza) supplemented with BIT 20%+P/S 1% and washed with PBS+P/S 1%. Cells were counted with Trypan Blue and resuspended in IMDM+BIT 20%+P/S 1% at a concentration of 50,000 cells/mL. A total of 5,000 CD34^+^ cells (100 µL) were seeded in 2.4 mL of MethoCult H4100® (Stem Cell Technologies) supplemented with FBS 30%, EPO 3 U/ml, 2-Mercaptoethanol 0.1 mM (Invitrogen, Merelbeke, Belgium), L-Glutamine 2 mM (Lonza), P/S 1%, SCF (Stem cell factor) 50 ng/mL (PeproTech, Neuilly-Sur-Seine, France) and with conditioned medium of the 5637 cell-line. TKIs were added from a stock solution of 10 mM in DMSO to the medium at a final concentration of 1 or 5 µM and cells were incubated for 14 days at 37°C under a 5% CO_2_ atmosphere. Colony forming cells (CFCs) were then counted.

### Long Term Culture-initiating Cell Assay

Absolute frequencies of LTC-ICs in cell suspensions recovered after a 48-hour cell culture incubation with TKIs at a concentration of 1 or 5 µM, or in control medium, were determined by limiting dilution analysis over MS-5 feeder cells. Briefly, the MS-5 feeder cell line was cultured in RPMI 1640 with 10% FBS. Cells were irradiated at 50 Gy and then plated in 96-well plates at 20,000 cells per well in 100 µL long-term culture (LTC) medium consisting of α-MEM supplemented with 8% horse serum, 8% fetal bovine serum, 0.2 mM glutamine, 100 U/mL penicillin and 100 µg/mL streptomycin (all from Lonza), 0.2 mM inositol (Sigma-Aldrich), 0.1 mM 2-mercaptoethanol. Within a week, thawed CD34^+^ cells were plated in limiting dilution in another 100 µL of LTC medium and maintained at 33°C in a 100% humidified atmosphere containing 5% CO_2_, with weekly half-medium change. After 6 weeks, medium was carefully aspirated from each well, followed by the addition of 200 µL of fully supplemented MethoCult. After an additional 2 weeks, wells were scored for the presence or absence of hematopoietic colonies, and the frequency of LTC-ICs was calculated using L-calc software (Stem Cell Technologies).

### Cell Cycle Analysis

Thawed CD34^+^ cells were counted with Trypan Blue and resuspended at a concentration of 1×10^6^ cells/mL. A total of 1×10^5^ cells/well (100 µL) was seeded in a 6-well plate, containing 2.4 mL of IMDM supplemented with BIT 20%, P/S 1%, SCF (100 ng/mL), TPO (50 ng/mL) and FLT-3 (100 ng/mL) (PeproTech). TKIs were added from a stock solution of 10 mM in DMSO at final concentrations of 1 µM or 5 µM. Flow cytometric cell cycle analyses of CD34^+^ cells cultivated during 48 hours with or without TKIs were performed using the CycleTEST™ Plus DNA Reagent Kit (BD Biosciences) as previously reported [18]. The percentage of cells in the different phases of the cell cycle was determined with Modfit software (BD Biosciences) on at least 20,000 acquired events. The percentage of cells in cycle was calculated as follows: percentage = ((S+G_2_/M cells)/Total cells)×100.

### VLA-4, VLA-5 and CXCR-4 Expression Analysis

Thawed CD34^+^ cells were counted with Trypan Blue and resuspended at a concentration of 1×10^6^ cells/mL. A total of 1×10^5^ cells/well was seeded in a 6-well plate, containing 2.4 mL of IMDM supplemented with BIT 20%, P/S 1%, SCF (100 ng/mL), TPO (50 ng/mL) and with FLT-3 (100 ng/mL). TKIs were added at final concentrations of 1 µM or 5 µM. After 48 hours of culture, human CD34^+^ cells were washed twice with PBS+FBS 3%+P/S 1% and were then incubated with FITC-conjugated anti-VLA-4 (BD Biosciences) or FITC-conjugated anti-VLA-5 (BD Biosciences) in combination with PE-conjugated anti-CXCR-4 (BD Biosciences) antibodies for 30 minutes at 4°C in the dark. Cells were then washed twice with PBS+FBS 3% and finally resuspended in pure PBS. Data acquisitions (at least 10,000 events) were performed on a FACSCanto II flow cytometer. Integrin density was expressed as the mean channel fluorescence ratio (MCFR) defined as the mean channel fluorescence (MCF) of CXCR-4 or integrin expression divided by MCF of fluorescence of the unstained control.

### Migration Assay

Migration assays were performed in 6.5 mm diameter 5 µm pore transwells. A total of 1×10^5^ CD34^+^ cells were plated in 100 µL of IMDM+BIT 20%+P/S 1%+SCF (100 ng/mL) in the upper chamber of the transwell. The bottom compartment was filled with IMDM supplemented with 20% BIT and 100 ng/mL stromal-derived factor-1 alpha (SDF-1α) (PeproTech). After incubation at 37°C during 4 hours, non-migrating and migrating cells were harvested by two standardized washes using PBS+FBS 3%+P/S 1%. Non-migrating and migrating cells were counted by flow cytometry using Trucount Tubes (BD Biosciences) after staining with an APC-conjugated anti-CD34 antibody. The percentage of non-migrating and migrating cells was calculated relative to the total number of harvested cells.

### Adhesion Assay

Thawed CD34^+^ cells were counted with Trypan Blue and resuspended at a concentration of 1×10^6^ cells/mL. An aliquot of 1.5×10^5^ cells/well were seeded in a 12-well plate containing 1 mL IMDM supplemented with BIT 20%, P/S 1%, SCF (100 ng/mL), TPO (50 ng/mL) and FLT-3 (100 ng/mL). TKIs were added from a stock solution of 10 mM in DMSO at final concentrations of 1 µM or 5 µM. After 48 hours of culture, human CD34^+^ cells were washed twice with PBS+P/S 1%.

Adhesion assays were performed in a 12-well plate. Wells were first coated with retronectin (Takara Bio Inc., Shiga, Japan) at a concentration of 9 µg/cm^2^ during two hours at 37°C. Supernatant was aspirated and wells were then incubated with PBS+BSA 1%+P/S 1% for 30 minutes at room temperature. Wells were finally washed twice with PBS+Hepes 2%+P/S 1%.

A total of 150,000 CD34^+^ cells resuspended in IMDM+BIT 20%+P/S 1% were added in each well and incubated for 90 minutes at 37°C. Supernatants were collected in polypropylene tubes. Adherent cells were detached by using the non-enzymatic cell dissociation buffer (Sigma) and collected in new polypropylene tubes. Cells were finally stained with an APC-conjugated anti-CD34 antibody and counted by flow cytometry using Trucount Tubes. The percentage of adherent cells was calculated relative to the total number of harvested cells.

### Transplantation into NOD/SCID/IL2rγ (Null) Mice

Six hours before CD34^+^ cell injection, NOD/SCID/IL-2Rγ (null) (NSG) mice (The Jackson laboratory, Bar Harbor, USA) were irradiated with 2.5 Gy TBI using a ^137^Cs source. Human CD34^+^ cells were thawed in IMDM+FBS 10%+P/S 1% and washed in PBS+P/S 1%. Cells were counted with Trypan Blue and resuspended in PBS at a concentration of 3×10^6^ cells/mL (6×10^5^ cells/200 µL). Mice were inoculated intravenously with 6×10^5^ CD34^+^ cells. Gavage with TKIs or a placebo was started at day 0. Imatinib was dissolved in sterile water and administrated at a dose of 150 mg/kg/day (50 mg/kg every morning and 100 mg/kg every evening) while nilotinib was prepared in 0.5% hydroxypropylmethyl cellulose (HPMC, Sigma) aqueous solution containing 0.05% Tween 80 and given at a concentration of 75 mg/kg/day (37.5 mg/kg every morning and evening). After 42 days, mice were sacrificed. Bone marrow cells from the two femurs were collected in sterile RPMI+FBS 10%+P/S 1%. Cells were counted with an HORIBA ABX® automatic cell counter (ABX Hematology, Montpellier, France). Cells were stained with anti-human CD45 (BD Biosciences) and anti-mouse CD45 (BD Biosciences) antibodies in order to determine the percentage of human chimerism by FACS analysis. Data acquisition was performed on a FACSCanto II flow cytometer on at least 20,000 mononuclear cells.

### Ethics Statement

All experiments using NSG mice were carried out in strict accordance with the recommendations in the Guide for the Care and Use of Laboratory Animals of the National Institutes of Health. The protocol was approved by the Committee on the Ethics of Animal Experiments of the University of Liège (Permit Number: 712). Mice were maintained in top-filtered cages in a standard animal facility and provided sterilized food and water *ad libitum*. Sterilized water supplemented with Baytril® 1% (Bayer HealthCare, Diegem, Belgium) was given from 3 days before to the end of the experiment. Water was change every 2–3 days. All euthanasia were performed under isoflurane anesthesia, and all efforts were made to minimize suffering.

### Statistical Analyses

Statistical analyses were performed with the GraphPad® Prism 5.00 Software. The paired Student’s T test was used to assess the impact of TKI on human cord blood CD34^+^ cells *in vitro*. Percentages and numbers of human cells in NSG mice were compared with the unpaired Student’s T test.

## Results

### Imatinib and Nilotinib Inhibited c-Kit Receptor Phosphorylation in a Dose-dependent Manner

**Figure 1:**
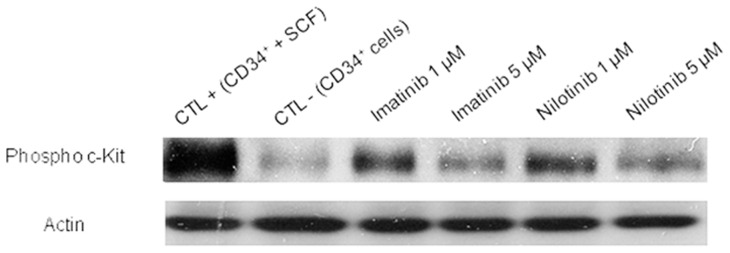
Inhibitory effects of TKIs on c-Kit phosphorylation in human CD34^+^ cord blood HSCs. The phosphorylated c-Kit receptor was detected by western blot after a 48-hour culture in presence of either imatinib or nilotinib. Representative picture from 4 independent experiments (n = 4).

We have first determined the inhibitory effect of TKIs on the c-Kit receptor cell signaling by western blot (n = 4). Human CD34^+^ cord blood HSCs were cultured in cytokine-supplemented medium for 48 hours with or without TKIs, washed and then lysed. Proteins were extracted on ice and dosed for western blotting analysis. The c-Kit phosphorylation levels in human CD34^+^ cells were decreased in a dose-dependent manner. Indeed at the highest concentration, both imatinib and nilotinib decreased dramatically the band intensity of phospho-c-Kit. No differences were seen between imatinib and nilotinib at a concentration of 1 µM and 5 µM (*Figure 1*).

### Both Imatinib and Nilotinib Inhibited Formation of Precursor Colony-forming Cells

**Figure 2:**
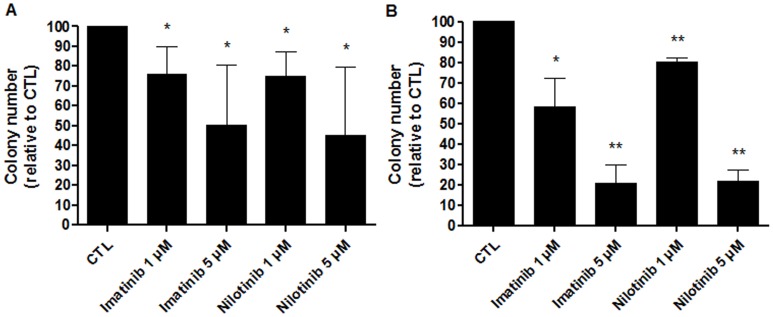
TKIs dramatically decrease CFC formation. (**A**)**:** Influence of a 48-hour pre-culture in the presence of either imatinib or nilotinib on CFC formation. (**B**)**:** CFC generation in CFC assays supplemented with TKIs. Results are expressed as mean percentages relative to control experiments without TKIs ± SD. n = 4, *p<0.05, **p<0.005 versus CTL, Student’s paired t tests. CTL: control condition without TKIs.

To determine whether imatinib and nilotinib could inhibit the generation of hematopoietic precursors, colony-forming cell (CFC) assays were carried out. In a first set of experiments (n = 4), CD34^+^ cells were cultured for 48 hours with or without TKIs, washed and then plated in cytokine-supplemented MethoCult for 14 days. Both imatinib and nilotinib significantly inhibited CFC formation. Indeed, imatinib, at a concentration of 1 or 5 µM, decreased CFC formation by a mean ± SD of 24.91±14.05% (*p = *0.0415) and 49.66±30.19% (*p = *0.0461) respectively, while nilotinib, at the same concentrations, reduced CFC numbers by 25.15±12.36% (*p = *0.0268 and *p = *0.8173 in comparison to imatinib) and 54.81±34.39% (*p = *0.0498 and *p = *0.1314 in comparison to imatinib), respectively (*Figure 2A*).

Next, cord blood CD34^+^ cells were seeded in MethoCult supplemented with or without imatinib or nilotinib at a final concentration of 1 or 5 µM for 14 days. As observed in the first series of experiments, TKIs significantly diminished CFC generation. Imatinib reduced colony formation by 41.67±14% (*p = *0.0356) at a concentration of 1 µM and 79.42±9.309% (*p = *0.0045) at 5 µM. Nilotinib decreased CFC formation by 19.64±1.90% (*p = *0.0031 and *p = *0.0898 in comparison to imatinib) and 78.28±5.27% (*p = *0.0015 and *p = *0.6733 in comparison to imatinib), respectively (n = 3, *Figure 2B*).

### Imatinib and Nilotinib did not Decrease Absolute Frequencies of LTC-ICs

**Figure 3:**
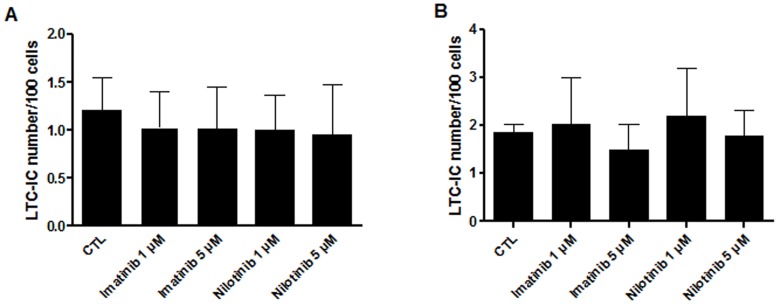
Neither imatinib nor nilotinib induce a decrease of LTC-IC frequency. (**A**)**:** Influence of a 48-hour pre-culture with imatinib or nilotinib at a final concentration of 1 or 5 µM on LTC-IC frequencies. (**B**)**:** LTC-IC generation with TKIs in a 6-week LTC-IC assay. Results are expressed as mean ± SD. n = 3, Student’s paired t tests. CTL: control condition without TKIs.

The capacity of TKIs to inhibit the differentiation of primitive hematopoietic progenitors was first assessed in long-term cultures. In a first set of experiments (n = 3), human cord blood CD34^+^ cells were incubated for 48 hours in the presence/absence of imatinib or nilotinib at a final concentration of 1 and 5 µM and then seeded in 96-well plates for LTC-IC assays without any TKIs. No significant differences were seen in the absolute frequencies of LTC-IC in each condition (*Figure 3A*).

In a second set of experiments (n = 3), CD34^+^ cells were directly plated for LTC-IC assays and incubated in the presence/absence of TKIs at a final concentration of 1 or 5 µM. Inhibitors were also added at all weekly half-medium change. As observed above, neither imatinib nor nilotinib decreased absolute frequencies of LTC-ICs (*Figure 3B*).

### Entry into Cell Cycle of Human Cord Blood CD34^+^ Cells was Impaired in the Presence of Imatinib and Nilotinib

**Figure 4:**
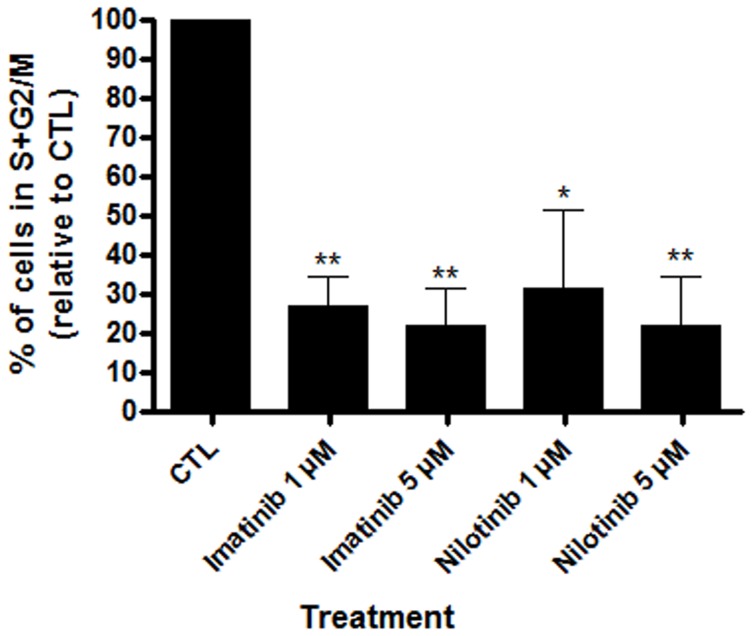
Colony formation in presence of tyrosine kinase inhibitors were decreased due to the inhibition of cell cycle entry. Human cord blood CD34^+^ were cultured for 48 hours in presence/absence of either imatinib or nilotinib and then stained with propidium iodide using the CycleTEST™ Plus DNA Reagent Kit. Results are expressed as mean percentages relative to control experiments without TKIs ± SD. n = 3, *p<0.05, **p<0.01 versus CTL, Student’s paired t tests. CTL: control condition without TKIs.

We assessed the impact of a 48-hour culture in the presence of imatinib or nilotinib on cord blood CD34^+^ cell proliferation. HSC proliferation was markedly reduced in the presence of imatinib 1 µM (73.2±4.5%; n = 3; *p = *0.003) or nilotinib 1 µM (68.4±11.4%; n = 3; *p = *0.026 and *p = *0.5620 in comparison to imatinib), and even more with the presence of 5 µM of imatinib (*p = *0.005) or nilotinib (*p = *0.008 and *p = *0.6746 in comparison to imatinib): HSC proliferation was decreased by 78.25±9.624% and 63.49±12.40% respectively (n = 3) (*Figure 4*).

### Expression of VLA-4 and VLA-5, but not the CXCR-4, Cell Surface Receptors were Decreased after a 48 h Cell-culture with Imatinib and Nilotinib

**Figure 5:**
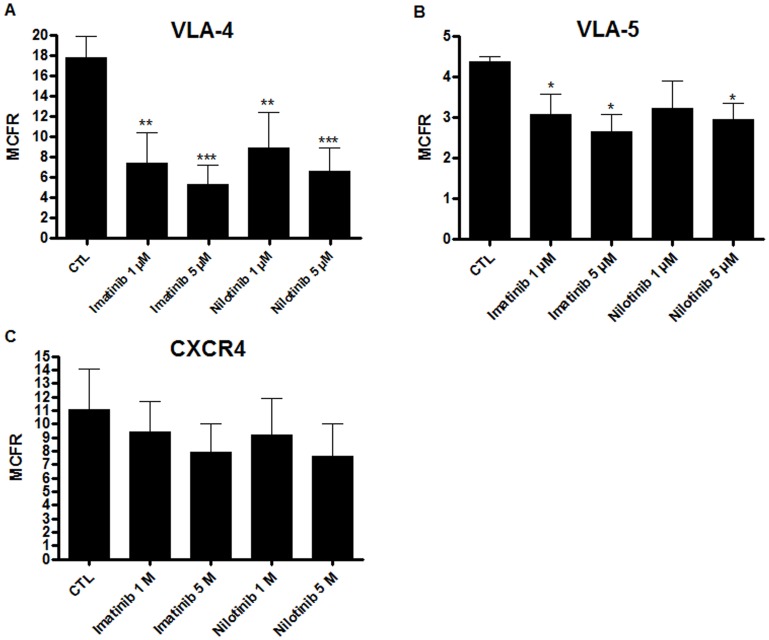
Cell surface expression of VLA-4 and VLA-5 were decreased after a 48 h cell-culture with TKIs while CXCR-4 expression was not affected. Influence of 48-hour pre-culture in presence of either imatinib or nilotinib on **(**A**):** VLA-4 **(**B**):** VLA-5 and **(**C**):** CXCR-4. Results are expressed as the mean channel fluorescence ratio (MCFR) ± SD. n ≥3, *p<0.05, **p<0.01, ***p<0.001 versus CTL, Student’s paired t tests. CTL: control condition without TKIs.

Homing of hematopoietic stem cells is a critical step for the success of allo-HSCT. In this process, three key-players have been identified: VLA-4, VLA-5 and CXCR-4 [19], [20]. Mean channel fluorescence ratio (MCFR) of the expression of these receptors on the cell surface of human cord blood CD34^+^ cells was determined by flow cytometry after a cell culture containing TKIs (or not). Imatinib significantly decreased expression of VLA-4 by a mean ± SD of 10.457±3.058 (*p* = 0.0032) and 12.520±1.872 (*p*<0.0001) at a concentration of 1 and 5 µM, respectively. Nilotinib at the same concentration induced the same effect by decreasing MCFR values by a mean ± SD of 8.920±3.472 (*p* = 0.0088) and 11.247±2.336 (*p* = 0.0008), respectively (n = 3) (*Figure 5A*).

VLA-5 expression was also decreased in the presence of both imatinib and nilotinib. Indeed, MCFR values were decreased by a mean ± SD of 1.307±0.5103 (*p = *0.0318) and 1.724±0.4219 (*p* = 0.0121) for imatinib at 1 and 5 µM, respectively. Nilotinib decreased VLA-5 MCFR values by a mean ± SD of 1.724±0.6833 (*p* = 0.0733) and 1.424±0.3931 (*p* = 0.0135) at the same concentration respectively (n = 3) (*Figure 5B*).

While both VLA-4 and VLA-5 expression were significantly decreased by TKIs, CXCR-4 cell surface expression was not affected upon 48-hour cell culture in the presence of imatinib or nilotinib (n = 6) (*Figure 5C*).

### Adhesion to Retronectin of Cord Blood CD34^+^ Cells was not Modified by 1 µM Imatinib and Nilotinib

**Figure 6:**
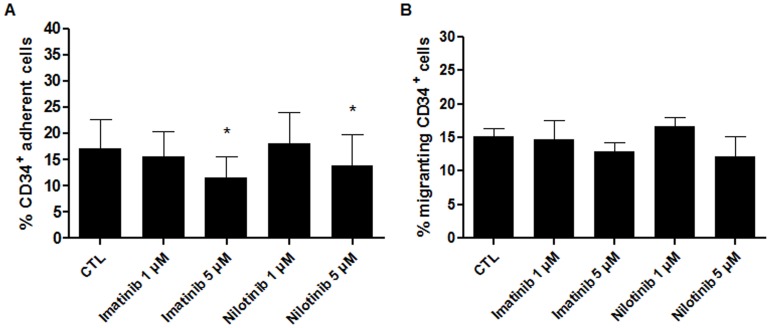
Influence of TKIs on adhesion and migration of human cord blood CD34^+^ cells. (**A**)**:** Adhesion of CD34^+^ cells to retronectin was significantly reduced in presence of the highest doses of TKIs. Results are expressed as the percentage of adherent cells ± SD. n = 10, *p<0.05 versus CTL, Student’s paired t tests. (**B**)**:** Migration of human HSCs toward a SDF-1α gradient was not modified by a 48 hour pre-culture period in the presence of TKIs. Results are expressed as the percentage of migrating cells ± SD. n = 4, Student’s paired t tests. CTL: control condition without TKIs.

As we observed a decreased in the expression of VLA-4 and VLA-5 in the presence of TKIs, we asked the question whether this lower expression affected the function of these receptors. We thus performed adhesion assays to retronectin with CD34^+^ cells cultured for 48 hours with TKIs. Imatinib 1 µM did not alter adhesion to retronectin (*p = *0.6186) while imatinib 5 µM decreased it by 5.69±4.134% (*p* = 0.0135) (n = 10). Similarly, nilotinib had no significant effect on adhesion at 1 µM, while at 5 µM it significantly reduced adhesion by 3.51±5.933% was observed (*p = *0.0432) (n = 10) (*Figure 6A*).

### A 48-hour Incubation with TKIs did not Affect the Capacity of Human CD34^+^ Cells to Migrate through a SDF-1α Gradient

No significant changes were observed in CXCR-4 expression in the presence of TKIs. To investigate the functional impact of these observations, migration assays of CD34^+^ HSCs were performed. CD34^+^ cells cultivated for 48 hours in the presence of TKIs were seeded in the upper chamber of a transwell containing SCF-supplemented medium. The lower chamber was filled with medium containing SDF-1α. Migration through the filter was allowed during 4 hours at 37°C. No significant difference were observed between cells cultivated with TKIs in comparison to the control (n = 4) (*Figure 6B*).

### Daily Dosing of Either Imatinib or Nilotinib did not Affect Repopulating Activity in NSG Mice

**Figure 7:**
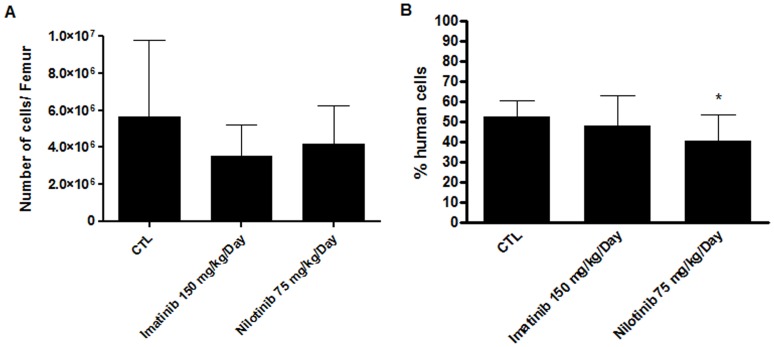
Effects of TKIs in a mouse model of transplantation. (**A**)**:** Daily doses of TKIs for 42 days did not affect bone marrow cellularity. Results are expressed as the mean number of cells per femur ± SD. n ≥8, Student’s unpaired t tests. (**B**)**:** Effect of continuous administration of TKIs on bone marrow chimerism. Imatinib did not affect the percentage of human cells in the bone marrow while nilotinib slightly decreased it compared to the control group. Results are expressed as the mean percentage ± SD. n ≥8, *p = 0.0314 versus CTL, Student’s unpaired t tests. CTL: control condition without TKIs.

Twenty-five sublethally irradiated NSG mice (in 3 independent experiments) were injected intravenously with 6×10^5^ human CD34^+^ cells and treated orally with placebo, imatinib 150 mg/kg/day or nilotinib 75 mg/kg/day for 42 days starting on day 0. No death occurred before the end of the experiments. Bone marrow cellularity was similar in the three groups. Numbers of cells/femur were (expressed as mean ± SD) 5.66±4.14×10^6^ in control mice, 3.55±1.67×10^6^ in mice treated with imatinib (*p = *0.1982) and 4.17±2.74×10^6^ in nilotinib-treated mice (*p* = 0.3731) (*Figure 7A*). Bone marrow chimerism was analyzed by flow cytometry. No significant differences were seen between mice treated placebo (52.5±2.7%; n = 9) or with imatinib (47.7±5.3%; n = 8; *p = *0.4130), while engraftment of human CD34^+^ cells was slightly decreased (40.6±4.4%; n = 8; *p = *0.0314) in mice treated with nilotinib (*Figure 7B*).

## Discussion

The aim of this project was to assess the impact of TKIs on hematopoietic stem cell function engraftment. Retrospective studies have suggested that treatment with TKIs before allo-HSCT did not preclude the outcome of engraftment and did not increase transplant-related toxicity [21]–[23]. Prospective studies showed that early administration of TKIs after allo-HSCT at a dose intensity comparable to that used in primary therapy seem to be safe [24] and can result in favorable long-term survival [12]. But the vast majority of these patients did not take TKIs during the engraftment period to prevent graft delay or failure. However, in the setting of non-myeloablative allo-HSCT for blast-crisis CML or Ph^+^ ALL, a 2–4 week TKI discontinuation may expose patients to early relapses. These data prompted us to evaluate the inhibitory effects of imatinib and nilotinib on the proliferation, differentiation and engraftment capacities of human cord blood CD34^+^ HSC.

The effects of TKIs were first assayed on LTC-ICs by limiting dilutions. Our data show that neither imatinib nor nilotinib had a significant impact on primitive progenitors. Indeed, as described before, LTC-IC frequencies were not affected by a 48 hour pre-culture or a 6-week incubation with TKIs [25], confirming previous findings that primitive HSC are less sensitive to TKIs than committed progenitors, possibly related to an enhanced presence of efflux drugs transporters in primitive HSCs [26].

We also evaluated the effects of TKIs on CFCs. Unlike LTC-IC frequencies, CFC growth was significantly decreased in culture containing either imatinib or nilotinib. Moreover, their inhibitory effects were permanent since a 48-hour pre-culture with TKIs is sufficient to decrease significantly the CFC generation definitively. Two hypotheses could explain these observations: (a) increased apoptosis or (b) inhibition of cell cycle entry. Since we and others have previously showed that imatinib do not increase the apoptosis of committed progenitors [18], [27], we investigated the proliferation of human CD34^+^ cells in the presence of TKIs. Our data demonstrate a significant decrease in HSC proliferation. These observations could be explain by the inhibition of others tyrosine kinases by imatinib and nilotinib. Indeed, *Bartolovic et al.* have shown that imatinib exerts growth inhibitory effect on normal CD34^+^ cells by the inhibition of SCF/c-kit pathway [27]. Moreover, studies on the effects of nilotinib on bone cells in Ph^+^ patients receiving nilotinib for treatment of CML have demonstrated that nilotinib potently inhibited osteoblast proliferation through inhibition of the platelet-derived growth factor (PDGFR). Furthermore, inhibition of c-Abl could contribute to the growth inhibition of CFCs by TKIs since antisense strategies have demonstrated that inhibition of c-Abl leads to the accumulation of CD34^+^ cells in G_0_/G_1_ and to inhibition of CFU-GM formation [28], [29]. Our results confirm also the results of Jorgensen and colleagues which showed that the predominant effect of imatinib and nilotinib on CD34^+^ CML cells is anti-proliferative rather than pro apoptotic. Indeed, the anti-proliferative effect of TKIs on Ph^+^ CD34^+^ cells is mainly caused by the inhibition of BCR-ABL [30].

Because VLA-4, VLA-5, and CXCR-4 play a major role in the homing of HSCs, we investigated the effect of TKIs on the expression of these surface receptors by flow cytometry. Despite our previous findings that the expression of VLA-4, VLA-5, and CXCR-4 of CD133^+^ cells was not modified by imatinib [18], a significant decrease in the expression of VLA-4 and VLA-5 was observed with either imatinib or nilotinib. However, no significant differences in CXCR-4 expression on CD34^+^ cells were seen. These apparent discrepancies could be explained by the cell source since in our previous publication [18], CD133^+^ cells isolated from peripheral blood of mobilized healthy volunteers were investigated while, in this study, CD34^+^ cells from cord blood were used in all experiments. Indeed, despite a higher VLA-4 and VLA-5 expression, cord blood CD34^+^ cells exhibit a lower CXCR-4 cell surface expression and a higher capacity to regenerate LTC-IC per competitive repopulating unit (CRU) [31] than on peripheral blood HSC cell surface [32], [33]. These differences in homing-related molecule expression could explain our discrepancies in the adhesion and migration behavior of cord blood CD34^+^. We then tested whether the decreased expression of VLA-4 and VLA-5 affected the capacity of CD34^+^ cells to adhere to retronectin *in vitro*. Adhesion was not affected by imatinib or nilotinib at physiological concentrations (1 µM) but decreased at higher doses (5 µM). CD34^+^ cell migration towards a SDF-1α gradient was not affected by TKIs. Inverse relationships between migration and adhesion capacities have often been observed. In HSCs, higher cell cycle activity is related with stronger adherence and decreased motility [34], [35]. However, because TKIs inhibit CD34^+^ cell proliferation, their effect on hematopoietic cell adhesion and migration appears to be independent of cell cycle activity. Additional studies will be necessary to investigate the impact of imatinib or nilotinib on tyrosine kinases implicated in adhesion and migration, such as the focal adhesion kinase or the related kinase PYK2 that is expressed in CD34^+^ cells [36].

Finally, we assessed the impact of TKIs on engraftment in a xenotransplantation model. Numbers of cells in the bone marrow of the femurs were similar in mice treated with placebo, imatinib or nilotinib. Moreover, no significant differences were seen in the percentages of bone marrow human CD45^+^ cells between mice treated with imatinib or placebo. However, the engraftment of human HSCs was slightly decreased in mice treated with nilotinib. This might be explained by the high daily dose of nilotinib (75 mg/kg/day) used in these experiments. Our results are comparable to those of our previous study [18] and with those of Hoepfl and colleagues, who demonstrated that imatinib (25 mg/kg twice daily) has no significant influence on hematopoietic engraftment in a syngeneic mouse bone marrow transplantation model [37].

On the basis of our data, combining non-myeloablative conditioning with TKIs for Ph^+^ ALL patients in order to maximize the graft-versus-leukemia effect could be possible with both nilotinib and imatinib.
